# Acute and 28-day repeated-dose oral toxicity of a synthetic 3-hydroxyindolin-2-one derivative in BALB/c mice

**DOI:** 10.1016/j.toxrep.2026.102354

**Published:** 2026-10-15

**Authors:** Nakibul Islam Mondal, Chitra Vellapandian

**Affiliations:** Department of Pharmacology, SRM College of Pharmacy, Faculty of Medicine and Health Sciences, SRM Institute of Science and Technology, Kattankulathur, Chengalpattu, Tamil Nadu, 603203, India

**Keywords:** 3-hydroxyindolin-2-one derivative, Acute & Subacute oral toxicity, BALB/c mice, Preclinical safety evaluation, Histopathology

## Abstract

Preclinical safety evaluation of newly synthesized bioactive molecules is a crucial step in the drug development process. The acute and subacute oral toxicity of the synthesized 3-Hydroxyindolin-2-one derivative, 7-bromo-3-hydroxy-3-(2-oxo-2-(p-tolyl)ethyl)indolin-2-one (4b), was evaluated in BALB/c mice using a sequential-dose acute oral toxicity/tolerability assessment and a 28-day repeated-dose study. For the acute oral assessment, compound 4b was administered sequentially at 2000, 1000, and 300 mg/kg body weight, followed by a 14-day observation period. Mortality was observed at 2000 and 1000 mg/kg, whereas no mortality was observed at 300 mg/kg, and subacute toxicity was determined by oral doses of 15, 30, and 60 mg/kg per day for 28 days. To determine the body weight, hematological parameters, biochemical parameters, gross pathological examination, and histopathological evaluation of major organs were performed for the synthesized compound. No mortality was observed at 300 mg/kg during the acute observation period or during the 28-day repeated-dose study, and no treatment-related gross pathological changes were observed at the examined doses. Several statistically significant differences and deviations from the reference intervals were observed among individual hematological and biochemical parameters following repeated administration. However, these changes did not demonstrate a consistent dose-response pattern across sexes and were not accompanied by concordant clinical, gross pathological, or histopathological findings. Histopathological evaluation of selected organs in the vehicle-control and high-dose groups showed well-preserved tissue architecture, with no treatment-related lesions identified in the examined tissues. Together, these results indicate that the synthesized compound 4b caused no mortality or clear treatment-related hematological, biochemical, gross pathological, or histopathological effects at the administered doses.

## Highlights

• No mortality was observed at 300 mg/kg or during 28-day repeated dosing.
• Selected-organ histopathology: no treatment-related lesions observed.
• Individual hematological and biochemical changes lacked a consistent treatment-related pattern.
• Histopathological examination: no lesions or organ damage observed.

## 1 Introduction

The alarming emergence and spread of antimicrobial resistance (AMR) have been identified as the most significant global health threat, and there is an urgent need to identify new therapeutic agents that are more effective and safer [1]. A family of heterocyclic compounds that has received much attention in the medicinal chemistry field is the 3-hydroxyindolin-2-one (isatin) derivatives, due to their broad range of biological activities, such as antioxidant, antifungal, anti-inflammatory, antibacterial, antiviral, and anticancer properties [2], [3], [4], [5]. Several modifications have been carried out in the isatin nucleus to improve the biological activity, pharmacokinetic properties, and limitations of some existing therapeutic agents [6]. Recent studies have shown that brominated and substituted 3-hydroxyindolin-2-one derivatives have strong antimicrobial activity against susceptible and resistant bacterial strains [7]. Hence, there is a growing interest in developing isatin-based derivatives with effective therapeutic effects. The molecules were designed for synthesis based on their previous antimicrobial and cytotoxic activity [8]. Several studies have demonstrated that structural modification of the 3-hydroxyindolin-2-one (isatin) scaffold is an effective strategy for improving its pharmacological properties and expanding its therapeutic potential. The introduction of suitable substituents at different positions of the isatin nucleus has resulted in derivatives with enhanced biological activity, improved target selectivity, and favorable physicochemical characteristics [9]. Consequently, brominated 3-hydroxyindolin-2-one derivatives have attracted great attention because of their broad spectrum of pharmacological activities, including anti-inflammatory, antiviral, antibacterial, antioxidant, and anticancer effects [10]. Building on these findings, 7-bromo-3-hydroxy-3-(2-oxo-2-(p-tolyl)ethyl)indolin-2-one (4b) was synthesized, and the present study was undertaken to characterize its acute and 28-day repeated-dose oral toxicity in BALB/c mice. Rodent models are broadly used in toxicological studies because of their genetic similarity to humans, well-characterized physiology, reproducible responses, and established use in safety and efficacy assessment [11]. Among these strains, BALB/c mice are particularly suitable for evaluating the toxicity of novel drug candidates due to their genetic background and stability for pharmacological research [12]. OECD test guidelines provide standardized approaches for evaluating acute and repeated-dose toxicity. OECD TG 425 describes an up-and-down procedure for acute oral toxicity, whereas OECD TG 407 addresses repeated-dose oral toxicity over a 28-day period [13], [14]. In the present study, the acute experiment was conducted as a sequential-dose acute oral toxicity/tolerability assessment referred by the general methodological principles of OECD TG 425, rather than a fully compliant OECD TG 425 up-and-down study. The 28-day repeated-dose study was designed with reference to OECD TG 407 and included body-weight monitoring, hematological and biochemical analysis, gross pathological examination, and histopathological assessment of the selected organs. Our earlier research revealed that 7-bromo-3-hydroxy-3-(2-oxo-2-(p-tolyl)ethyl)indolin-2-one (4b) demonstrated promising antibacterial activity against pathogenic bacteria, indicating its potential as a lead antimicrobial candidate. However, its *in vivo* safety profile has not been reported. Thereafter, extensive toxicological evaluation is essential before further pharmacological development. This study was designed to investigate the acute and 28-day repeated dose oral toxicity of synthesized compound 4b in BALB/c mice. The acute assessment was performed by toxicological observations, body-weight monitoring, hematological and biochemical analysis, gross pathological examination, and histopathological assessment of selected organs. Acute oral toxicity helps to identify the adverse effects of a single exposure and provides preliminary information on the toxicological effects of a test compound. Subacute toxicity studies assess the systemic effects of repeated oral administration over a defined period and can provide dose-related toxicological information to support dose selection for subsequent pharmacological and toxicological investigations.

## 2 Materials and methods

### 2.1 Synthesis and characterization of 7-bromo-3-hydroxy-3-(2-oxo-2-(p-tolyl)ethyl)indolin-2-one

The compound 7-bromo-3-hydroxy-3-(2-oxo-2-(p-tolyl)ethyl)indolin-2-one (4b) was synthesized and discussed in our previous study, and FTIR, ^1^H NMR, ^13^C NMR, and HRMS spectroscopy analysis characterized the compound. Thereafter, the compound was purified using 1% cold ethanol and then washed with n-hexane three times to remove impurities [8].

### 2.2 Experimental conditions and approval from the animal ethical committee

The animals were procured from Mass Biotech Chengalpattu, Tamil Nadu, India (CPCSEA Regn. No.: *2084/PO/RcBt/S/19/CPCSEA)* and utilized both Male and female, eight-week-old BALB/c mice 25–30 g. After acclimatization, the animals were kept according to standard laboratory conditions for one week, with a temperature of 22 ± 3 °C, humidity of 50 ± 5%, and a 12 h light/dark cycle. Male and female animals were kept separately in groups of 5 per cage and provided free access to feed and water during the experimental period. The animal experiments were conducted following approval from the Institutional Animal Ethics Committee of CPCSEA (IAEC/401/2026) on 07 February 2026. The 28-day repeated-dose oral toxicity study was designed with reference to OECD TG 407. The acute experiment was conducted as a sequential-dose acute oral toxicity/tolerability assessment, with OECD TG 425 used only as a methodological reference; it was not conducted as a fully compliant OECD TG 425 up-and-down procedure, and animals were euthanised for histopathological, hematological, and biochemical parameters. During the experiment, animals were regularly observed for signs such as weight loss, prostration, organ dysfunction, breathing difficulties, abnormal behavior, excessive salivation, and convulsions. After the study completion, the animals were euthanized by inhalational overdose of isoflurane (5% in an induction chamber), and death was confirmed by permanent cessation of respiration and heartbeat.

### 2.3 Sequential-dose acute oral toxicity/tolerability assessment

A sequential-dose acute oral toxicity/tolerability assessment was conducted in BALB/c mice. The procedure was performed by the general principles described in OECD TG 425; however, this experiment was not conducted as a fully compliant OECD TG 425 up-and-down procedure. Mice were randomly selected, marked for identification, and acclimatized for at least 1 week before administration. Animals were fasted overnight before dosing and had free access to water. Compound 4b was administered orally at sequential dose levels. An initial dose of 2000 mg/kg body weight was administered to two animals followed by a subsequent dose of 1000 mg/kg body weight to one animal, if mortality observed [13]. Based on mortality at higher dose, a further lower dose of 300 mg/kg body weight was administered to two animals. A separate vehicle-control animal received 3% DMSO. Animals were closely observed for mortality and clinical signs of toxicity during the first 30 min, 1 h, and 3 h, and 48 h following administration and subsequently at regular intervals for 14 days. Observations included changes in general behavior, locomotor activity, posture, respiration, salivation, piloerection, convulsions, food intake, and other signs of toxicity [15]. Body weight was monitored during the observation period. At the end of the 14-day observation period, the animals were euthanized following the approved euthanasia procedure. A gross necropsy was subsequently performed, during which the external body surface and major organs within the thoracic and abdominal cavities were examined macroscopically for visible abnormalities. Blood samples were also collected from the retro-orbital plexus using a capillary tube for hematological and biochemical analyses [16].

### 2.4 28-day repeated-dose oral toxicity

Forty healthy eight-week-old BALB/c mice, comprising 20 males and 20 females, were allocated into four groups of 10 animals each, with five males and five females per group. Animals were assigned to the experimental groups using simple randomization. Animals were housed five per cage by sex and treatment group, and individual animals were the experimental units for individual-level toxicity measurements. One group served as the vehicle control, while the remaining three groups received compound 4b orally at 15, 30, and 60 mg/kg/day for 28 consecutive days [14]. Compound 4b was administered by oral gavage using 3% DMSO (vehicle control) at a dosing volume of 0.1–0.3 mL/kg according to the animal body weight [15]. The control animals received the corresponding vehicle at the same dosing volume. Dosing was performed each morning. The required quantities of compound 4b were formulated in the range of 0.3–1.8 mg/mL to obtain the concentrations required for the three dose levels. The formulations were prepared by dissolution followed by vortexing and administered immediately to the animals receiving the preparation. No analytical verification of formulation concentration, homogeneity, or stability was performed in the present study. Formulations were prepared immediately before administration by dissolution followed by vortexing and were administered without prolonged storage. Before initiation of treatment, all animals underwent a general health examination and were monitored daily for one week during the acclimatization period. Throughout the 28-day treatment period, animals were observed daily for mortality, toxicity, and any abnormal findings were recorded. Observations were performed at approximately the same time each day in relation to the scheduled dosing time. Observations included changes in general appearance, posture, locomotor activity, gait, respiration, salivation, piloerection, pallor, diarrhea, and other abnormal behavioral or physical signs, which were monitored and recorded. Body weight was recorded before initiation of treatment (Day 0) and subsequently at 7-day intervals throughout the 28-day treatment period (Day 7, 14, 21, and 28), while food and water consumption were measured at 20 g per cage on a daily basis and water consumption at 15–20 mL per cage on a daily basis throughout the 28-day study in reference to OECD TG 407. Then calculated mean daily intake per animal for each group to assess potential treatment-related effects on nutritional status and exposure. Functional observations were limited to assessment of motor activity and selected reflex responses, including corneal and righting reflexes [16], [17]. A comprehensive standardized functional-observation battery was not conducted. Daily toxicological observations were performed throughout the 28-day treatment period; however, a separate formal functional-observation battery was not conducted. At the end of the 28-day treatment period, the animals were fasted overnight and euthanized on day 29 using 5% isoflurane inhalation. Prior to euthanasia, blood samples were collected from the animals for hematological and biochemical analyses. A detailed gross necropsy was subsequently performed, and the major organs were examined macroscopically for any visible abnormalities. The brain, heart, lungs, stomach, colon, liver, spleen, kidneys, pancreas, testes, ovaries, and uterus were collected and preserved in 10% neutral buffered formalin for histopathological examination. Organ weights were not recorded during the present study; therefore, absolute and relative organ-weight data are not reported. Histopathological examination was subsequently performed to assess potential treatment-related microscopic alterations.

### 2.5 Hematology and biochemical analysis

Blood was collected in EDTA-containing tubes from animals for hematological and biochemical analyses. Complete blood count (CBC) parameters, including hemoglobin (Hb), white blood cell (WBC) count, platelets, red blood cell (RBC), neutrophils, lymphocytes, packed cell volume (PVC), eosinophils, monocytes, mean corpuscular hemoglobin (MCH), mean corpuscular hemoglobin concentration (MCHC), mean corpuscular volume (MCV), and basophils were determined and interpreted against standard and clinical reference interval [18], [19]. Observed biochemical parameters included blood glucose, urea, creatinine, aspartate aminotransferase (AST/SGOT), uric acid, alanine aminotransferase (ALT/SGPT), total bilirubin, alkaline phosphatase, HDL cholesterol, total protein, albumin, total cholesterol, globulin, triglyceride, LDL, and VLDL. Electrolyte parameters, such as sodium, chloride, potassium, and bicarbonate, were also measured and compared with the standard ranges [20], [21].

### 2.6 Histopathology

Following euthanasia, the brain, heart, lungs, liver, kidneys, spleen, pancreas, stomach, colon, and reproductive organs were collected and preserved in freshly prepared 10% neutral buffered formalin for 24 h at 4 °C. Histopathological evaluation was performed on five male and five female mice from the vehicle-control group and five male and five female mice from the high-dose group (60 mg/kg/day). Thus, a total of 20 animals were evaluated histopathologically in the 28-day study. The intermediate-dose groups (15 and 30 mg/kg/day) were not subjected to histopathological examination. Following fixation, the tissues were routinely processed through graded concentrations of ethanol for dehydration, cleared in xylene, and infiltrated and embedded in paraffin wax. The paraffin blocks were sectioned at 3–5 µm thickness using a rotary microtome. The sections were mounted on glass slides, deparaffinized, rehydrated, and stained with hematoxylin and eosin (H&E) using standard procedures. Multiple sections from each collected organ were examined microscopically. The stained sections were examined by an experienced histopathologist who was blinded to the treatment allocation during microscopic evaluation. The stained tissue sections were examined by light microscopy for treatment-related morphological alterations and abnormalities in cellular and tissue architecture [18], [22].

### 2.7 Statistical analysis

All experimental data were presented as mean ± standard error of the mean (SEM), and Statistical analysis was performed using two-way analysis of variance (ANOVA), followed by Tukey’s multiple-comparisons test, using GraphPad Prism software version 8.0.2. A *P* value < 0.05 was considered statistically significant [23]. Statistical comparisons were made with the corresponding vehicle-control group. ANOVA-based statistical estimation of LD₅₀ was not performed; the acute toxicity outcome was determined from the observed survival/mortality pattern at the sequential dose levels.

## 3 Results

### 3.1 Synthesis and characterization of 7-bromo-3-hydroxy-3-(2-oxo-2-(p-tolyl)ethyl)indolin-2-one (4b)

7-bromoisatin (0.5 g) was reacted with 3-acetophenone (0.9 g) in the presence of a few drops of diethylamine as a catalyst, and 10 mL of ethanol was added to the mixture as a solvent; the mixture was stirred at room temperature to produce compound 4b. The synthesized compound was purified by washing with 1% cold ethanol and then again washed to remove impurities using *n*-hexane. The synthesised product was confirmed by analytical characterization, such as FT-IR, ^1^H NMR, ^13^C NMR, and HR-MS, as discussed in our previous study (Synthesized data will be provided on request to the author).

### 3.2 Sequential-dose acute oral toxicity/tolerability assessment

The synthesized compound 7-bromo-3-hydroxy-3-(2-oxo-2-(p-tolyl)ethyl)indolin-2-one (4b) was evaluated using a sequential-dose acute oral toxicity/tolerability assessment. An initial dose of 2000 mg/kg body weight was administered, at which mortality was observed during the observation period. Furthermore, a subsequent dose of 1000 mg/kg body weight, mortality observed. Based on the mortality observed at these higher dose, the dose was further reduced to 300 mg/kg body weight. Throughout the observation period, at 300 mg/kg, no abnormal behavior, physical changes, mortality or treatment-related adverse effects were recorded in any of the animals for 14 days. The mice exhibited normal behavior, food intake, and healthy physical appearance. At the end of the study, after 14 days of observation, the animals were euthanized, and necropsy was performed. Gross pathological examination revealed no abnormalities in organ structure or morphology. No mortality was observed at 300 mg/kg during the 14-day observation period listed in Table 1. This dose therefore served as the empirical basis for selecting the lower doses for the subsequent 28-day repeated-dose study.

**Table 1:** Acute oral toxicity/tolerability assessment of compound 4b in BALB/c mice.

**Dose (mg/kg)**	**Number of animals**	**Mortality**	**14-day outcome**
2000	2	Observed	Mortality
1000	1	Observed	Mortality
300	2	Not observed	Survived 14-day observation
Vehicle control (3% DMSO)	1	Not observed	Vehicle control

Sequential-dose acute oral toxicity assessment of compound 4b in BALB/c mice and mortality observed during the 14-day observation period. The table presents the administered dose levels, number of animals, mortality outcome, and survival status during the 14-day observation period. Compound 4b was administered orally at sequential dose levels of 2000, 1000, and 300 mg/kg, with 3% DMSO used as the vehicle control.

### 3.3 Hematology and biochemical analysis

Acute oral administration of compound 4b at 300 mg/kg was associated with statistically significant differences from the vehicle-control group for several hematological and biochemical parameters (Table 2), and several measured values differed from the reference intervals. In the hematological assessment, RBC, WBC, MCHC, and hemoglobin were close to the stated physiological ranges, whereas platelet count, MCV, MCH, neutrophils, eosinophils, lymphocytes and monocytes were outside the reference intervals. These findings indicate that the hematological changes should be interpreted in relation to the vehicle-control values and the reference intervals rather than being considered uniformly normal. In the biochemical assessment, total bilirubin, AST/SGOT, ALT/SGPT, total protein, globulin, blood glucose, urea, total cholesterol, and triglycerides showed no major changes within the stated reference intervals (Table 2). ALP was above the reference range, whereas albumin, creatinine, and uric acid were below their respective reference intervals. Several biochemical parameters showed statistically significant differences from the vehicle-control group; these changes were not interpreted solely on the basis of statistical significance or deviation from reference intervals. Accordingly, the acute findings at 300 mg/kg did not demonstrate a clear, coherent pattern of treatment-related hematological, hepatic, renal, or metabolic toxicity under the conditions tested.

**Table 2:** Hematological and biochemical parameters of mice following acute oral administration of compound 4b.

**Hematology**				**Biochemistry**			
**Parameters**	**Control**	**Tested**	**Reference range**	**Parameters**	**Control**	**Tested**	**Reference range**
**RBC** (×10^6^/µL)	5.35 ± 0.08	6.91 ± 0.04****	6.9–12	**Bilirubin total** (mg/dL)	0.87 ± 0.03	0.64 ± 0.04 ^ns^	0.3–1.2
**WBC** (×10^3^/µL)	11.13 ± 0.06	9.80 ± 0.03****	2.5–14.5	**SGOT** (U/L)	189.31 ± 0.51	163.31 ± 0.51****	55–352
**Hb** (g/dL)	13.28 ± 0.10	13.22 ± 0.04 ^ns^	M: 13.5–17.0 F: 12.0–15.5	**SGPT** (U/L)	102.43 ± 0.39	66.43 ± 0.39****	41–131
**Platelet** (×10^3^/µL)	5.17 ± 0.08	10.62 ± 0.11****	1.5–4.00	**Total protein** (g/dL)	5.58 ± 0.21	4.52 ± 0.12****	4.5–6.5
**PCV** (%)	46.67 ± 0.12	39.70 ± 0.14****	M: 40–52 F: 38–45	**Alkaline phosphatase** (U/L)	198.10 ± 0.09	152.10 ± 0.09****	53–141
**MCV** (fL)	62.66 ± 0.09	65.81 ± 0.08****	76–96	**Albumin** (g/dL)	3.88 ± 0.04	1.90 ± 0.06****	3.5–5.2
**MCH** (pg)	17.66 ± 0.35	23.22 ± 0.11****	27–32	**Globulin** (g/dL)	1.84 ± 0.05	2.59 ± 0.06**	0.4–3.2
**MCHC** (g/dL)	28.46 ± 0.12	32.46 ± 0.12****	32–36	**Blood glucose (R)** (mg/dL)	83.26 ± 0.28	121.26 ± 0.28****	70–140
**Neutrophils** (%)	48.43 ± 0.05	3.22 ± 0.11****	50–70	**Urea** (mg/dL)	26.20 ± 0.34	23.20 ± 0.34****	10–40
**Eosinophils** (%)	2.08 ± 0.04	0.22 ± 0.03****	0.5–5.0	**Creatinine** (mg/dL)	0.63 ± 0.06	0.53 ± 0.04 ^ns^	0.6–1.2
**Basophils** (%)	0.00 ± 0.00	0.00 ± 0.00 ^ns^	0.0–1.0	**Uric acid** (mg/dL)	0.91 ± 0.06	0.75 ± 0.03 ^ns^	0.91–1.92
**Lymphocytes** (%)	43.35 ± 0.20	96.67 ± 0.22****	48.8–83	**Total cholesterol** (mg/dL)	68.11 ± 0.11	54.11 ± 0.11****	42–88
**Monocytes** (%)	5.8 ± 0.11	0.00 ± 0.00****	0.4–6.1	**Triglyceride** (mg/dL)	108.08 ± 0.09	67.08 ± 0.09****	< 200

Data presented as mean ± SEM. Statistical analysis was performed using Tukey's multiple comparisons test and two-way analysis of variance (ANOVA). Statistical significance was defined as P < 0.05. In the tables, **** indicates P < 0.0001, *** indicates P < 0.001, ** indicates P < 0.01, and * indicates P < 0.05; ns indicates P ≥ 0.05. After the acute oral toxicity study, the following hematological and biochemical parameters were assessed. The test group received compound 4b (300 mg/kg body weight), and the vehicle control group received 3% DMSO. Data are presented as mean ± SEM. Hb, hemoglobin; RBC, red blood cell count; WBC, white blood cell count; MCV, mean corpuscular volume; MCHC, mean corpuscular hemoglobin concentration; PCV, packed cell volume; MCH, mean corpuscular hemoglobin; SGOT, serum glutamic-oxaloacetic transaminase (AST); SGPT, serum glutamic-pyruvic transaminase (ALT); and R, random blood glucose.

### 3.4 28-day repeated-dose oral toxicity

Compound 4b was administered orally for 28 consecutive days at doses of 15, 30, and 60 mg/kg/day, designated as the low-, mid-, and high-dose groups, respectively. These dose levels were selected based on the acute toxicity findings, in which no mortality was observed at 300 mg/kg during the 14-day observation period, while mortality was observed at the higher tested doses of 1000 and 2000 mg/kg. The study referenced the OECD 407 guideline (OECD 407 compliance table shown in Table S2). There were no treatment-related mortalities; no pathological abnormalities were observed in either male or female animals. Interestingly, body weights also increased over time in all experimental groups (Fig. 1), but there was no significant difference between the tested drug-treated and control animals. No significant differences in food or water consumption were observed between treated and control groups, indicating that compound 4b did not affect feeding behavior or hydration. No treatment-related changes in body weight, food consumption, water consumption, or gross pathological findings were observed following repeated oral administration of 4b at doses up to 60 mg/kg/day.

**Fig. 1:**
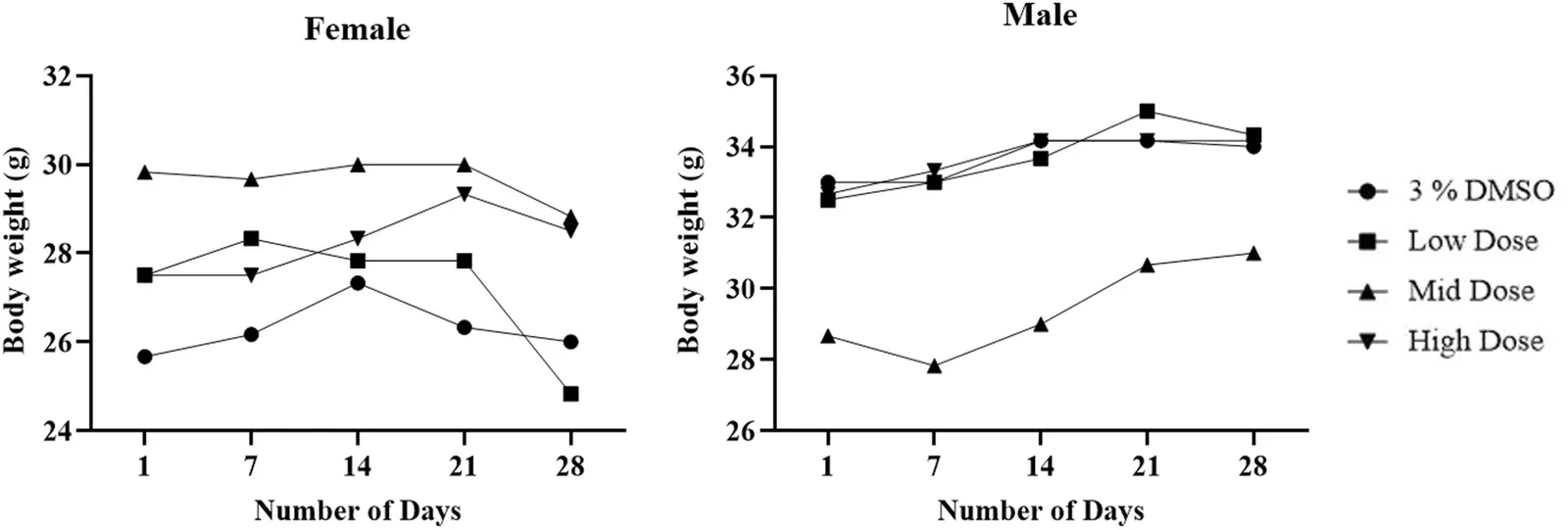
Effect of synthesized compound (4b) at different doses on body weight of mice in subacute toxicity studies. The data (n = 5) are presented as mean ± SEM.

### 3.5 Hematological and biochemical parameters

The hematological parameters after repeated administration of 4b (15, 30, and 60 mg/kg) are given in Table 3. Several statistically significant differences were observed for individual parameters compared with the vehicle control; however, these changes did not associate with a consistent dose-response pattern, and no corresponding pathological findings were seen in either male or female BALB/c mice during the treatment. Compared with the reference intervals in Table 3, several parameters deviated, particularly MCV, MCH, neutrophils, lymphocytes, and platelet count. MCV and MCH values were below the reference intervals in both male and female mice across all treatment groups. Neutrophil percentage were below the reference intervals in both male and female mice at all treatment doses, whereas lymphocyte percentages were above the reference interval in male mice at 15, 30, and 60 mg/kg and in female mice at 15 and 30 mg/kg. Platelet counts were above the reference interval in both male and female mice across all treatment groups. These deviations were not characterized by a consistent dose-dependent pattern across both sexes. Therefore, the findings were interpreted in conjunction with the vehicle-control values, statistical comparisons, biochemical observations, and other toxicity endpoints rather than solely on the basis of the reference intervals. RBC counts were within the reference interval in both male and female treatment groups, whereas the control values were below the reference interval. Hemoglobin values were showed no changes compared with the reference intervals in most groups; the male values at 15 and 30 mg/kg were above the reference interval, and the male value at 60 mg/kg was below the reference interval; female values remained within the reference interval. PCV values were generally within the reference intervals; the male value at 60 mg/kg was below the reference interval. MCHC values were generally within the reference interval, except for the male group at 15 mg/kg, which was slightly below the reference interval. Total WBC, differential leukocyte and platelet counts showed several statistically significant differences compared with the vehicle-control group; however, these changes did not demonstrate a consistent dose-response pattern across males and females. These hematological findings did not demonstrate a clear treatment-related toxicological pattern under the conditions tested. Table 4 represents the renal biochemical parameters. Blood glucose concentrations were within the reference interval in both male and female control and treatment groups; statistically significant differences were observed between the treatment groups and their respective vehicle controls. Blood glucose concentration did not show a consistent dose-dependent increase or decrease across the treatment groups.

**Table 3:** Hematological parameters of male and female BALB/c mice following subacute oral administration of 4b.

**Parameters**	**Control**		**15 mg/kg**		**30 mg/kg**		**60 mg/kg**		***Reference range***
	**(M)**	**(F)**	**(M)**	**(F)**	**(M)**	**(F)**	**(M)**	**(F)**	
**RBC** (×10^6^/µL)	4.85 ± 0.11	5.45 ± 0.01	9.05 ± 0.07****	7.10 ± 0.02****	6.93 ± 0.05****	8.41 ± 0.08****	8.25 ± 0.25****	7.55 ± 0.13****	6.9–12
**WBC** (×10^3^/µL)	7.17 ± 0.03	13.26 ± 0.20	10.17 ± 0.04****	10.53 ± 0.12****	9.49 ± 0.02****	6.18 ± 0.06****	5.67 ± 0.12****	9.80 ± 0.15****	4.00–11.00
**Hb** (g/dL)	10.21 ± 0.16	13.33 ± 0.11	15.69 ± 0.03****	14.12 ± 0.04****	15.39 ± 0.08****	14.49 ± 0.09****	12.54 ± 0.11****	14.06 ± 0.10****	M: 13.5–17.0 F: 12.0–15.5
**Platelet** (×10^3^/µL/ mm^3^)	1.39 ± 0.08	5.24 ± 0.06	7.06 ± 0.05****	5.73 ± 0.04****	4.27 ± 0.09****	9.68 ± 0.19****	8.75 ± 0.30****	5.7 ± 0.18****	1.5–4
**Neutrophils** (%)	33.29 ± 0.01	48.44 ± 0.29	29.36 ± 0.15****	14.30 ± 0.02****	20.09 ± 0.10****	16.49 ± 0.10****	5.28 ± 0.35****	14.49 ± 0.10****	50–70
**Lymphocytes** (%)	65.91 ± 0.03	43.56 ± 0.21	67.73 ± 0.09****	79.70 ± 0.08****	76.29 ± 0.08****	79.47 ± 0.34****	91.49 ± 0.12****	79.63 ± 0.06****	48.8–83
**Eosinophils** (%)	0.51 ± 0.01	2.12 ± 0.16	1.86 ± 0.07****	0.27 ± 0.04****	0.15 ± 0.04 ^ns^	0.31 ± 0.08****	2.48 ± 0.11****	0.23 ± 0.03****	0.5–5.0
**Monocytes** (%)	0.30 ± 0.02	5.92 ± 0.17	0.89 ± 0.09****	5.56 ± 0.12 ^ns^	3.66 ± 0.04****	3.70 ± 0.26****	0.33 ± 0.03 ^ns^	5.59 ± 0.13 ^ns^	0.4–6.1
**Basophils** (%)	0.01 ± 0.01	0.03 ± 0.05	0.003 ± 0.005 ^ns^	0.009 ± 0.01 ^ns^	0.01 ± 0.02 ^ns^	0.01 ± 0.01 ^ns^	0.02 ± 0.03 ^ns^	0.02 ± 0.03 ^ns^	0.0–1.0
**PCV** (%)	30.40 ± 0.07	46.65 ± 0.23	49.79 ± 0.12****	41.35 ± 0.07****	44.76 ± 0.12****	43.82 ± 0.20****	37.72 ± 0.13****	41.46 ± 0.40****	M: 40–52 F: 38–45
**MCV** (fL)	62.6 ± 0.02	62.61 ± 0.22	56.11 ± 0.17****	53.24 ± 0.06****	52.4 ± 0.20****	52.23 ± 0.36****	47.27 ± 0.15****	53.47 ± 0.18****	76–96
**MCH** (pg)	20.76 ± 0.04	17.82 ± 0.23	17.56 ± 0.12****	18.19 ± 0.05****	20.25 ± 0.21****	17.20 ± 0.21****	15.42 ± 0.22****	18.33 ± 0.14****	27–32
**MCHC** (g/dL)	33.20 ± 0.04	28.62 ± 0.11	31.34 ± 0.05****	34.11 ± 0.05****	35.25 ± 0.15****	32.67 ± 0.15****	33.37 ± 0.24 ^ns^	34.29 ± 0.20****	32–36

Data presented as mean ± SEM (n = 5 per group). Statistical analysis was performed using Tukey's multiple comparisons test and two-way analysis of variance (ANOVA). Statistical significance was defined as P < 0.05. In the tables, **** indicates P < 0.0001, *** indicates P < 0.001, ** indicates P < 0.01, and * indicates P < 0.05; ns indicates P ≥ 0.05. Hematological parameters were examined in both male (M) and female (F) BALB/c mice following repeated oral administration of compound 4b at 15, 30, and 60 mg/kg body weight/day for 28 consecutive days, and the control group received the vehicle (3% DMSO). Data are expressed as mean ± SEM (n = 5 animals/sex/group). Hb, hemoglobin; RBC, red blood cell count; WBC, white blood cell count; PCV, packed cell volume; MCH, mean corpuscular hemoglobin; MCV, mean corpuscular volume; MCHC, mean corpuscular hemoglobin concentration.

**Table 4:** Serum biochemical parameters (renal function) in both male and female BALB/c mice following repeated oral (subacute) administration of 4b.

**Parameters**	**Control**		**15 mg/kg**		**30 mg/kg**		**60 mg/kg**		***Reference range***
	**(M)**	**(F)**	**(M)**	**(F)**	**(M)**	**(F)**	**(M)**	**(F)**	
**Blood glucose (R)**	135.22 ± 0.23	83.36 ± 0.47	95.13 ± 0.15****	100.07 ± 0.06****	98.15 ± 0.14****	89.16 ± 0.17****	98.36 ± 0.35****	101.36 ± 0.35****	70–140
**Urea**	24.16 ± 0.19	26.50 ± 0.44	23.08 ± 0.11****	28.31 ± 0.30****	29.27 ± 0.25****	24.19 ± 0.24****	26.27 ± 0.24****	31.26 ± 0.30****	10–40
**Creatinine**	0.51 ± 0.02	0.49 ± 0.01	0.40 ± 0.02 ^ns^	0.4 ± 0.03 ^ns^	0.43 ± 0.04 ^ns^	0.44 ± 0.04 ^ns^	0.52 ± 0.02 ^ns^	0.51 ± 0.04 ^ns^	0.6–1.2
**Uric acid**	0.91 ± 0.01	0.95 ± 0.06	0.89 ± 0.07 ^ns^	0.66 ± 0.15 ^ns^	0.93 ± 0.02 ^ns^	0.56 ± 0.07 ^ns^	0.92 ± 0.01 ^ns^	0.62 ± 0.01 ^ns^	0.91–1.92

Data presented as mean ± SEM (n = 5 per group). Statistical analysis was performed using Tukey's multiple comparisons test and two-way analysis of variance (ANOVA). Statistical significance was defined as P < 0.05. In the tables, **** indicates P < 0.0001, *** indicates P < 0.001, ** indicates P < 0.01, and * indicates P < 0.05; ns indicates P ≥ 0.05. Serum biochemical parameters (renal function) were evaluated in male (M) and female (F) BALB/c mice following repeated oral administration of 4b at 15, 30, and 60 mg/kg body weight/day for 28 consecutive days, and the control group received the vehicle (3% DMSO). Data are expressed as mean ± SEM (n = 5 animals/sex/group). R, random blood glucose. Blood glucose, urea, creatinine, and uric acid concentrations are expressed in mg/dL.

Table 4 presents serum urea, creatinine, and uric acid concentrations. Urea and uric acid values showed no changes compared with the reference interval in both males and females. Creatinine values were below the reference interval in both male and female control and treatment groups. Creatinine and uric acid values did not differ significantly from the respective vehicle controls and showed no consistent dose-dependent pattern. In the absence of a consistent treatment-related pattern and corresponding gross or histopathological findings in the examined organs, the biochemical data did not provide any clear evidence of treatment-related renal toxicity. There is no deviation in the serum urea and creatinine levels, and the lack of a consistent dose-related pattern does not indicate a clear treatment-related alteration in the evaluated renal biochemical parameters. Therefore, renal function should not be considered normal solely on these biochemical findings, particularly because creatinine and some uric acid values were below the reference intervals. The renal findings did not demonstrate a clear treatment-related pattern indicative of renal toxicity under the conditions tested.

Several parameters differed significantly from the vehicle-control groups (Table 5). No dose-dependent increase in liver transaminases was observed. SGOT and SGPT values remained consistent with the reference interval in both males and females in all groups; male values generally decreased with increasing dose, while female values did not show a consistent dose-related pattern. In all groups, total bilirubin levels were within the reference interval, with no statistically significant differences compared with the respective vehicle-control groups. ALP values were above the reference interval in both male and female control and treatment groups. Total protein, albumin and globulin values remained within the reference interval in both male and female treatment groups. Several hepatic biochemical parameters differed significantly from the vehicle-control group; the changes did not show a consistent dose-dependent pattern, and some deviations from the reference intervals were also observed in the vehicle-control groups. The histopathological assessment of the examined organs and the biochemical findings did not provide clear evidence of treatment-related hepatic toxicity under the conditions tested.

**Table 5:** Hepatic biochemical parameters of mice for repeated oral administration of 4b.

**Parameters**	**Control**		**15 mg/kg**		**30 mg/kg**		**60 mg/kg**		***Reference range***
	**(M)**	**(F)**	**(M)**	**(F)**	**(M)**	**(F)**	**(M)**	**(F)**	
**Bilirubin total**	0.69 ± 0.01	0.90 ± 0.02	0.80 ± 0.04 ^ns^	0.90 ± 0.02 ^ns^	0.82 ± 0.04 ^ns^	0.9 ± 0.02 ^ns^	0.9 ± 0.02 ^ns^	0.83 ± 0.04 ^ns^	0.3–1.2
**SGOT**	199.2 ± 0.17	189.27 ± 0.28	176.05 ± 0.07****	189.13 ± 0.15 ^ns^	179.25 ± 0.22****	191.10 ± 0.12****	169.2 ± 0.26****	176.4 ± 0.45****	55–352
**SGPT**	101.13 ± 0.15	102.20 ± 0.24	93.30 ± 0.33****	102.26 ± 0.24 ^ns^	89.26 ± 0.25****	105.35 ± 0.38****	91.16 ± 0.15****	93.28 ± 0.32****	41–131
**Alkaline phosphatase**	169.09 ± 0.13	198.38 ± 0.41	183.15 ± 0.25****	179.36 ± 0.31****	191.12 ± 0.13****	210.17 ± 0.28****	170.12 ± 0.11****	193.25 ± 0.25****	53–141
**Total protein**	5.61 ± 0.01	5.80 ± 0.16	6.19 ± 0.12****	5.79 ± 0.17 ^ns^	6.92 ± 0.07****	5.72 ± 0.14 ^ns^	6.33 ± 0.20****	6.3 ± 0.24****	4.5–6.5
**Albumin**	2.70 ± 0.02	3.90 ± 0.04	4.32 ± 0.22****	3.91 ± 0.05 ^ns^	1.91 ± 0.04****	3.88 ± 0.06 ^ns^	3.57 ± 0.03****	4.32 ± 0.06 ^ns^	3.5–5.2
**Globulin**	2.90 ± 0.03	1.9 ± 0.02	1.75 ± 0.07****	1.86 ± 0.04 ^ns^	5.12 ± 0.04****	1.91 ± 0.05 ^ns^	2.51 ± 0.08 ^ns^	1.68 ± 0.11 ^ns^	0.4–3.2

Data presented as mean ± SEM (n = 5 per group). Statistical analysis was performed using Tukey's multiple comparisons test and two-way analysis of variance (ANOVA). Statistical significance was defined as P < 0.05. In the tables, **** indicates P < 0.0001, *** indicates P < 0.001, ** indicates P < 0.01, and * indicates P < 0.05; ns indicates P ≥ 0.05. Hepatic biochemical parameters were evaluated in both male (M) and female (F) mice following repeated oral administration of 4b at 15, 30, and 60 mg/kg body weight/day for 28 consecutive days, and the control group received the vehicle (3% DMSO). Data are expressed as mean ± SEM (n = 5 animals/sex/group). SGOT, serum glutamic-oxaloacetic transaminase (aspartate aminotransferase, AST); SGPT, serum glutamic-pyruvic transaminase (alanine aminotransferase, ALT); ALP, alkaline phosphatase; A/G ratio, albumin-to-globulin ratio. Bilirubin is expressed as mg/dL; SGOT, SGPT, and ALP as U/L; total protein, albumin, and globulin as g/dL; and A/G ratio is dimensionless.

Table 6 presents the serum lipid profile. HDL concentrations were below the reference interval listed in the table in both male and female control and treatment groups. These values were interpreted primarily relative to the vehicle-control groups rather than as evidence of a treatment-related abnormality based solely on the reference interval. Total cholesterol showed no changes relative to the reference interval in all groups, but male mice showed statistically significant increases at 15 and 30 mg/kg and a statistically significant decrease at 60 mg/kg compared with the male control group. LDL, triglyceride, and VLDL concentrations showed no major changes in both male and female groups compared with reference ranges. No changes were observed for triglycerides, HDL, or VLDL in both male & female groups. Several lipid parameters differed significantly from vehicle controls, particularly in male mice. The changes did not demonstrate a consistent dose-response relationship across sexes. The lipid findings did not demonstrate a consistent treatment-related pattern indicative of a major disturbance in lipid homeostasis during the 28-day treatment period.

**Table 6:** Serum lipid profile of mice following repeated oral doses of compound 4b.

**Parameters**	**Control**		**15 mg/kg**		**30 mg/kg**		**60 mg/kg**		***Reference range***
	**(M)**	**(F)**	**(M)**	**(F)**	**(M)**	**(F)**	**(M)**	**(F)**	
**Total cholesterol**	61.26 ± 0.22	63.33 ± 0.35	76.08 ± 0.10****	63.37 ± 0.32 ^ns^	86.25 ± 0.22****	63.25 ± 0.28 ^ns^	58.36 ± 0.40****	51.33 ± 0.35****	42–88
**Triglyceride**	79.11 ± 0.17	108.56 ± 0.48	102.18 ± 0.17****	108.07 ± 0.10 ^ns^	97.09 ± 0.15****	108.13 ± 0.12 ^ns^	84.25 ± 0.24****	91.2 ± 0.26****	42–155
**HDL Cholesterol**	15.26 ± 0.24	15.72 ± 0.07	19.09 ± 0.10****	15.76 ± 0.08 ^ns^	21.52 ± 0.14****	15.68 ± 0.14 ^ns^	14.53 ± 0.08****	12.65 ± 0.11****	35–126
**LDL**	29.94 ± 0.03	25.65 ± 0.07	36.44 ± 0.17****	25.68 ± 0.12 ^ns^	45.24 ± 0.12****	25.68 ± 0.27 ^ns^	26.73 ± 0.09****	20.13 ± 0.07****	12.0–39.6
**VLDL**	15.82 ± 0.02	21.69 ± 0.17	20.48 ± 0.19****	21.62 ± 0.15 ^ns^	19.48 ± 0.09****	21.64 ± 0.05 ^ns^	16.7 ± 0.12****	18.31 ± 0.09****	7.5–38.8

Data presented as mean ± SEM (n = 5 per group). Statistical analysis was performed using Tukey's multiple comparisons test and two-way analysis of variance (ANOVA). Statistical significance was defined as P < 0.05. In the tables, **** indicates P < 0.0001, *** indicates P < 0.001, ** indicates P < 0.01, and * indicates P < 0.05; ns indicates P ≥ 0.05. Serum lipid parameters were evaluated in mice following repeated oral administration of compound 4b at 15, 30, and 60 mg/kg body weight/day for 28 consecutive days, and the control group received the vehicle (3% DMSO). Data are expressed as mean ± SEM (n = 5 animals/sex/group). HDL-C, high-density lipoprotein cholesterol; LDL-C, low-density lipoprotein cholesterol; VLDL-C, very-low-density lipoprotein cholesterol. Total cholesterol, triglycerides, HDL-C, LDL-C, and VLDL-C are expressed in mg/dL.

Table 7 presents serum electrolyte concentrations. Sodium and potassium concentrations didn’t change relative to reference ranges in either the male or female group. Potassium concentrations also showed several statistically significant differences compared with the vehicle-control groups; no changes were observed relative to the reference interval. Chloride concentrations were below the reference ranges in both male and female control and treatment groups, whereas bicarbonate concentrations were above the reference ranges in both male and female control and treatment groups. These deviations were therefore not considered treatment-specific based solely on comparison with the reference intervals. Several electrolyte measurements showed statistically significant differences, the changes did not show a consistent dose-dependent pattern across sexes. No accompanying clinical signs or other findings indicative of disturbed electrolyte homeostasis were observed. Therefore, repeated oral administration of 4b did not cause major significant changes in electrolyte balance or acid-base homeostasis in the mice.

**Table 7:** Serum electrolyte levels in mice treated with compound 4b.

**Parameters**	**Control**		**15 mg/kg**		**30 mg/kg**		**60 mg/kg**		***Reference range***
	**(M)**	**(F)**	**(M)**	**(F)**	**(M)**	**(F)**	**(M)**	**(F)**	
**Sodium**	139.54 ± 0.19	141.23 ± 0.15	132.35 ± 0.20****	133.43 ± 0.08****	139.53 ± 0.08 ^ns^	141.41 ± 0.07 ^ns^	147.3 ± 0.09****	138.49 ± 0.10****	128–187
**Potassium**	4.59 ± 0.08	5.65 ± 0.07	4.12 ± 0.11****	4.50 ± 0.17****	4.57 ± 0.05 ^ns^	5.32 ± 0.05****	5.63 ± 0.11****	4.82 ± 0.18****	3.1–12.2
**Chloride**	99.18 ± 0.08	98.42 ± 0.07	95.46 ± 0.13****	100.15 ± 0.08****	98.51 ± 0.09****	97.49 ± 0.07****	98.52 ± 0.16****	97.49 ± 0.11****	107–139
**Bicarbonate**	27.14 ± 0.10	29.28 ± 0.02	26.29 ± 0.15****	28.22 ± 0.03****	26.30 ± 0.06****	25.26 ± 0.11****	27.28 ± 0.14 ^ns^	26.58 ± 0.29****	18–22

Data presented as mean ± SEM (n = 5 per group). Statistical analysis was performed using Tukey's multiple comparisons test and two-way analysis of variance (ANOVA). Statistical significance was defined as P < 0.05. In the tables, **** indicates P < 0.0001, *** indicates P < 0.001, ** indicates P < 0.01, and * indicates P < 0.05; ns indicates P ≥ 0.05. Serum electrolyte levels were evaluated in BALB/c mice following repeated oral administration of compound 4b at 15, 30, and 60 mg/kg body weight/day for 28 consecutive days, and the control group received the vehicle (3% DMSO) under the same experimental conditions. Data are expressed as mean ± SEM (n = 5 animals/sex/group). M, male; F, female. Sodium (Na⁺), potassium (K⁺), chloride (Cl⁻), and bicarbonate (HCO₃⁻) levels are expressed in mmol/L.

Several hematological and biochemical values differed from the reference intervals presented in Table 3, Table 4, Table 5, Table 6, Table 7, and statistically significant differences from the vehicle-control group were observed for selected parameters. These deviations were therefore not uniformly described as being within physiological ranges. Their toxicological relevance was assessed by considering the direction and magnitude of change, dose-response pattern, sex-related consistency, comparison with the vehicle-control group, and concordance with gross pathological and histopathological findings. Isolated deviations were observed; they did not collectively demonstrate a consistent treatment-related pattern indicative of organ toxicity under the conditions of the 28-day study.

### 3.6 Histopathological evaluation

Gross necropsy did not reveal any apparent treatment-related abnormalities in the examined organs of animals administered compound 4b at a dose of 15, 30, or 60 mg/kg/day for 28 days. Prior to euthanasia, blood samples were collected for hematological and biochemical analyses. The major organs were subsequently collected for histopathological examination. Histopathological evaluation was performed in the vehicle-control and high-dose groups. The intermediate-dose groups (15 and 30 mg/kg/day) were not subjected to histopathological evaluation, as no treatment-related changes were observed at the high dose. Histopathological evaluation was performed on the brain, lungs, heart, kidneys, liver, pancreas, spleen, stomach, colon, and reproductive organs of both male and female BALB/c mice following repeated oral administration of 60 mg/kg/day (high dose) of compound 4b for 28 days. No treatment-related lesions were observed in any of the examined animals (0/5 males and 0/5 females). Microscopic examination demonstrated normal structural features comparable to those observed in the control group, with no apparent treatment-related tissue damage, necrosis, fibrosis, or irreversible pathological changes in the examined organs. Organ weights were not measured in the present study and therefore could not be included as an endpoint. (shown in Table S1 and Figures S1 & S2 – refer to the supplementary file).

## 4 Discussion

This study provides preliminary toxicological information on compound 4b following acute and 28-day repeated oral administration in BALB/c mice. During the acute assessment, mortality was observed at the higher tested dose levels, but no mortality was observed at 300 mg/kg during the 14-day observation period. The 300 mg/kg dose was then used as an empirical basis for selecting the lower doses for the repeated-dose study. Importantly, the acute experiment was performed as a sequential-dose acute oral toxicity/tolerability assessment, referenced by the general methodological principles of OECD TG 425, but was not conducted as a fully compliant OECD TG 425 up-and-down procedure. Therefore, the present findings should not be interpreted as establishing an LD₅₀ value. The 28-day repeated-dose findings did not show a consistent treatment-related toxicological pattern across the endpoints examined. Statistically significant changes in some hematological and biochemical parameters were observed, but no similar dose-response relationship was evident between males and females, and gross pathology changes were related to the treatment. A few of the measured values also differed from the reference intervals. Thus, toxicological relevance was evaluated in vehicle-control values, dose-response relationships, sex-related variation, statistical comparisons, and concordance among toxicity endpoints rather than reference intervals alone. This combined interpretation is significant because deviation from a reference interval in an individual parameter, without a consistent treatment-related pattern and corroborating toxicity findings, should not be interpreted independently as evidence of synthesized compound-related toxicity. The hematological findings therefore require interpretation in the context of the toxicity profile rather than from individual parameters alone. The observed variations in erythrocytic indices, differential leukocyte parameters, and platelet counts were not associated with a consistent dose-dependent pattern or corresponding pathological findings. Thus, the present hematological information does not provide as evidence of a coherent treatment-related effect on hematopoietic function under the conditions tested [24]. Similarly, the observed variations in hepatic and renal markers were considered together with vehicle-control values, dose-response relationships, and the absence of corresponding pathological findings. The lack of a consistent treatment-related increase in hepatic or renal injury-associated parameters, together with the findings from gross examination and histopathology, does not indicate a clear treatment-related hepatic or renal toxicological effect under the conditions of the 28-day study. The lipid and electrolyte findings were also interpreted in the context of the pattern rather than individual statistical differences, some parameters differed from the reference intervals or showed statistical differences from vehicle controls, these changes were not characterized by a consistent dose-response relationship across sexes and were not accompanied by disturbed metabolic or electrolyte homeostasis findings. The histopathological findings provided additional context for interpretation of the biochemical observations. No treatment-related microscopic lesions were identified in the examined organs of the vehicle-control and high-dose groups, and the observed tissue architecture was comparable between the examined groups. However, these findings should be interpreted within the scope of the histopathological assessment, which was limited to selected organs and to the vehicle-control and high-dose groups but the absolute and relative organ weights were not recorded.

The present findings indicate that no consistent treatment-related toxicological pattern was identified within the scope and duration. This interpretation should not be taken to mean that all measured parameters remained within the physiological reference intervals or that long-term safety has been established. Longer-duration toxicity studies, pharmacokinetic characterization, genotoxicity assessment [25] and efficacy studies will be required to further characterize the toxicological and pharmacological profile of compound 4b.

## 5 Limitation

A limitation of the present study is that absolute and relative organ weights were not recorded during the 28-day toxicity experiment. Consequently, organ-weight changes could not be evaluated as an independent toxicity endpoint. In addition, histopathological examination was limited to selected major organs rather than the complete tissue panel recommended in OECD TG 407. Interpret the present histopathological findings within the scope of the organs examined. The present study did not analytically verify formulation concentration, homogeneity, or stability. The formulations were prepared immediately before administration by dissolution followed by vortexing; the absence of analytical formulation verification limits confirmation of the nominal concentrations and uniformity of the administered preparations. The assessment of potential treatment-related toxicity was based on body-weight changes, hematological and biochemical parameters, gross necropsy findings, and histopathological examination of the selected organs.

## 6 Conclusion

In conclusion, the synthesized 3-hydroxyindolin-2-one derivative, 7-bromo-3-hydroxy-3-(2-oxo-2-(p-tolyl)ethyl)indolin-2-one (4b), did not show mortality or clear treatment-related toxicity under the conditions of the present sequential-dose acute oral assessment and 28-day repeated-dose study in BALB/c mice. During the 28-day study, administration of 4b at a dose of 15, 30, and 60 mg/kg/day did not produce a consistent treatment-related pattern across the evaluated body-weight, hematological, biochemical, gross pathological, and examined histopathological endpoints. Several hematological and biochemical values differed from the reference intervals or vehicle controls; these changes did not demonstrate a consistent dose-response relationship. These findings provide preliminary toxicological information on compound 4b within the scope and duration of the present investigation and do not establish its overall safety. The acute assessment was informed by the general methodological principles of OECD TG 425 but was not a fully compliant OECD TG 425 up-and-down procedure and therefore did not establish an LD₅₀ value. Further longer-duration toxicity, pharmacokinetic, genotoxicity, and efficacy studies are required to further characterize the toxicological profile of compound 4b.

## CRediT authorship contribution statement

**Nakibul Islam Mondal:** Writing – original draft, Visualization, Validation, Formal analysis, Data curation, Conceptualization. **Chitra Vellapandian:** Writing – review & editing, Supervision, Investigation, Formal analysis.

## Declaration of Generative AI and AI-assisted technologies in the writing process

During the preparation of this work, the author(s) used Grammarly in order to improve grammatical errors during manuscript writing. After using this tool, the authors reviewed and edited the content as needed and take full responsibility for the published article.

## Declaration of Competing Interest

The authors declare that they have no known competing financial interests or personal relationships that could have appeared to influence the work reported in this paper.

## Data Availability

No data was used for the research described in the article.
