# Supplementary material for: Acute and 28-day repeated-dose oral toxicity of a synthetic 3-hydroxyindolin-2-one derivative in BALB/c mice
Source: Toxicol Rep. 2026 Oct 15;17:102354. doi: 10.1016/j.toxrep.2026.102354 (PMC13715401; doi:10.1016/j.toxrep.2026.102354)
Supplement: Supplementary file 1 [file mmc1.docx]

Supplementary Information (*SI*)

**Acute and 28-day repeated-dose oral toxicity of a synthetic 3-hydroxyindolin-2-one derivative in BALB/c mice**

Nakibul Islam Mondal^1^, Chitra Vellapandian^1^**^*^**

^1^Department of Pharmacology, SRM College of Pharmacy, Faculty of Medicine and Health Sciences, SRM Institute of Science and Technology, Kattankulathur, Chengalpattu, Tamil Nadu 603203, India.

**Corresponding author**

*Chitra Vellapandian

*E-mail:* [chitrav@srmist.edu.in](mailto:chitrav@srmist.edu.in)

|  | **Control (M)** | **Control (F)** | **60mg/kg (M)** | **60mg/kg (F)** |
| --- | --- | --- | --- | --- |
| **Brain** |  |  |  |  |
| **Lung** |  |  |  |  |
| **Heart** |  |  |  |  |
| **Colon** |  |  |  |  |
| **Stomach** |  |  |  |  |
| **Liver** |  |  |  |  |
| **Pancreas** |  |  |  |  |
| **Spleen** |  |  |  |  |
| **Kidney** |  |  |  |  |

**Figure S1:** Representative hematoxylin and eosin (H&E)-stained sections of major organs from BALB/c mice following 28-day oral administration of compound 4b. Representative sections from the vehicle-control and high-dose (60 mg/kg/day) groups are shown. No apparent treatment-related histopathological alterations were observed. Scale bar = 200 µm; original magnification = 10 X

**Abbreviations:** M: Male, F: Female; **Brain**- N: Neurons, GC: Glial cell, BV: Blood vessel, NP: Neuropil, P: Perineuronal space; **Lungs**- A: Alveoli, LN: Lymphatic nodule, B: Bronchiole, P: Type II pneumocyte, E: Epithelium, SE: Subepithelium, C: Capillary, BV: Blood vessel, PC: Pulmonary capillary, B: Bronchiole; **Heart**- CM: Cardiomyocyte, MCN: Muscle cell nucleus, ECL: Endothelial cell lining, NC: Nuclei of cardiomyocytes, CC: Coronary capillary, CM: Cardiac muscle Fibers, E: Endomysium; **Colon**- CL: Crypts of Lieberkühn, GC: Goblet cells, MM: Muscularis mucosae, S: Submucosa, SE: Subepithelium, CM: Colonic mucosa, L: Lumen; **Stomach**- GC: Gastric mucous neck cells, PC: parietal cells, GG: gastric glands, S: submucosa, ML: muscularis layer, GM: Gastric mucosa, SM: Submucosa; **Liver**- CV: Central vein, PT: portal triad, S: Sinusoids, H: Hepatocytes, BD: Bile duct, PV: Portal vein, N: Nucleus; **Pancreas**- L: Lobules, IL: Islet of Langerhans, ID: Intercalated duct, A: Acini, PA: Pancreatic acini, C: Capillaries, CS: Connective tissue septa, AC: Acinar cell, BV: Blood vessel, CT: Connective tissue, PG: Pancreatic gland; **Spleen**- C: Capsule, CA: Central arteriole, WP: White pulp, RP: Red pulp, GC: Germinal center, S: Sinuses, C: Capillaries, S: Sinusoids; **Kidney**- G: Glomeruli, BV: Blood vessels, PC: Proximal convoluted tubule, DC: Distal convoluted tubule, RP: Renal parenchyma, T: Tubules, CD: Collecting duct, TL: Tubular lumen.

|  | **Control** | **60mg/kg** |
| --- | --- | --- |
| **Testis (M)** |  |  |
| **Ovary (F)** |  |  |
| **Uterus (F)** |  |  |

**Figure S2: Micrograph photos of reproductive organs from control and high-dose groups**. H&E staining was used for the study, with 200 µm scale bar.

**Abbreviations:** M: Male; F: Female; **Testis**- T: Tubules, SC: Sertoli cells, S: Spermatogonia, ST: Spermatocytes, SD: Spermatids, SM: Smooth muscle; **Ovary**-TFS: Thick fibrous stroma, OP: Ovarian parenchyma, C: Capillaries, OS: Ovarian stroma; **Uterus**- UG: Uterine glands, CE: Columnar epithelium, C: Capillaries, EG: Endometrial glands, M: Myometrium.

**Table S1- Histologically examined lesions in the major organs**

| **Organ** | **Group** | **Lesions observed** | **Conclusion** |
| --- | --- | --- | --- |
| **Brain** | Control (M) | None | These findings indicate that the test compound did not produce any observable neurotoxicity or adverse effects on the brain in the high-dose group and the control. |
|  | Control (F) |  |  |
|  | 60 mg/kg (M) |  |  |
|  | 60 mg/kg (F) |  |  |
| **Lungs** | Control (M) | None | These findings indicate that the test compound did not produce any observable pulmonary toxicity or adverse effects on lung tissue in the high-dose group and the control. |
|  | Control (F) |  |  |
|  | 60 mg/kg (M) |  |  |
|  | 60 mg/kg (F) |  |  |
| **Heart** | Control (M) | None | These findings indicate that the test compound did not produce any observable cardiotoxicity or adverse effects on heart tissue in the high-dose group or the control group. |
|  | Control (F) |  |  |
|  | 60 mg/kg (M) |  |  |
|  | 60 mg/kg (F) |  |  |
| **Colon** | Control (M) | None | These findings indicate that the test compound did not produce any observable colon toxicity or adverse effects on colon tissue in the high-dose group and the control. |
|  | Control (F) |  |  |
|  | 60 mg/kg (M) |  |  |
|  | 60 mg/kg (F) |  |  |
| **Stomach** | Control (M) | None | These findings indicate that the test compound did not produce any observable gastrotoxic effects in the high-dose group and the control. |
|  | Control (F) |  |  |
|  | 60 mg/kg (M) |  |  |
|  | 60 mg/kg (F) |  |  |
| **Liver** | Control (M) | None | These findings indicate that the test compound did not produce any hepatotoxicity in the high-dose group and the control. |
|  | Control (F) |  |  |
|  | 60 mg/kg (M) |  |  |
|  | 60 mg/kg (F) |  |  |
| **Pancreas** | Control (M) | None | These findings indicate that the test compound did not produce any observable pancreatic toxicity or adverse effects on pancreatic tissue in the high-dose group or the control. |
|  | Control (F) |  |  |
|  | 60 mg/kg (M) |  |  |
|  | 60 mg/kg (F) |  |  |
| **Spleen** | Control (M) | None | These findings indicate that the test compound did not produce any observable adverse effects on spleen tissue in the high-dose group and the control group. |
|  | Control (F) |  |  |
|  | 60 mg/kg (M) |  |  |
|  | 60 mg/kg (F) |  |  |
| **Kidney** | Control (M) | None | These findings indicate that the test compound did not produce any observable nephrotoxicity or adverse effects on kidney tissue in the high-dose group or the control. |
|  | Control (F) |  |  |
|  | 60 mg/kg (M) |  |  |
|  | 60 mg/kg (F) |  |  |

**Table S2:** **Comparison of the present 28-day repeated-dose oral toxicity study with the principal recommendations of OECD Test Guideline 407.**

| **OECD TG 407 element** | **OECD 407 expectation/principle** | **Present study** | **Compliance/status** |
| --- | --- | --- | --- |
| **Test species** | Rodent species; commonly mice; other rodents may be used with justification | BALB/c mice, 8 weeks old, 25-30 g | Performed |
| **Sex** | Both sexes | 20 males + 20 females | Performed |
| **Number/group** | Appropriate number allowing meaningful evaluation | 5 males + 5 females/group | Performed |
| **Control** | Concurrent vehicle control | 3% DMSO vehicle control | Performed |
| **Dose groups** | three dose levels | 15, 30 and 60 mg/kg/day | Performed |
| **Route** | Appropriate repeated oral administration | Oral gavage | Performed |
| **Treatment duration** | 28 consecutive days | 28 consecutive days | Performed |
| **Randomization** | Animals allocated appropriately to groups | Simple randomization | Performed |
| **Clinical observations** | Regular/daily clinical observations | Daily observations for mortality and clinical signs | Performed |
| **Functional observations** | Functional observations during study | Motor activity and corneal/righting reflexes assessed; formal FOB not conducted | Partially performed |
| **Body weight** | Regular body-weight measurements | Days 0, 7, 14, 21 and 28 | Performed |
| **Food consumption** | Regular monitoring | Daily measurement per cage | Performed |
| **Water consumption** | Regular monitoring | Daily measurement per cage | Performed |
| **Hematology** | Appropriate hematological parameters | CBC and differential parameters assessed | Performed |
| **Clinical biochemistry** | Appropriate biochemical parameters | Hepatic, renal, lipid and electrolyte parameters assessed | Performed |
| **Urinalysis** | Where applicable/according to study design | Not performed | Not performed |
| **Necropsy** | Detailed gross pathological examination | Gross necropsy performed | Performed |
| **Organ weights** | Absolute and relative organ weights | Not recorded | Not performed |
| **Histopathology** | Histopathological examination of appropriate organs | Control and high-dose groups examined | Performed with limitation |
| **Lower-dose histopathology** | Additional groups examined when treatment-related effects are observed | Intermediate-dose groups not examined histologically | Not performed; justified by absence of treatment-related findings at high dose |
| **Tissue declaration processing** | Appropriate fixation, processing and staining | 10% neutral buffered formalin, paraffin embedding, 3-5 µm sections, H&E | Performed |
| **Ethical approval** | Institutional ethical oversight | IAEC/401/2026 | Performed |
| **Euthanasia** | Humane euthanasia | Isoflurane inhalation | Performed |
| **Study duration** | 28-day exposure | 28 days | Performed |
